# European Society of Paediatric Radiology Artificial Intelligence taskforce: a new taskforce for the digital age

**DOI:** 10.1007/s00247-022-05426-3

**Published:** 2022-06-22

**Authors:** Lene Bjerke Laborie, Jaishree Naidoo, Erika Pace, Pierluigi Ciet, Christine Eade, Matthias W. Wagner, Thierry A. G. M. Huisman, Susan C. Shelmerdine

**Affiliations:** 1 Department of Radiology, Section for Paediatrics, Haukeland University Hospital, Bergen, Norway; 2 Department of Clinical Medicine, University of Bergen, Bergen, Norway; 3 Paediatric Diagnostic Imaging and Envisionit Deep AI, Johannesburg, South Africa; 4 Department of Diagnostic Radiology, The Royal Marsden NHS Foundation Trust, London, UK; 5 Department of Radiology and Nuclear Medicine, Erasmus MC, Rotterdam, The Netherlands; 6 Department of Pediatric Pulmonology and Allergology, Erasmus MC, Sophia’s Children’s Hospital, Rotterdam, The Netherlands; 7 University of Exeter Medical School, Exeter, UK; 8 Department of Diagnostic Imaging, Division of Neuroradiology, The Hospital for Sick Children, Toronto, Canada; 9 Department of Medical Imaging, University of Toronto, Toronto, Ontario Canada; 10 Edward B. Singleton Department of Radiology, Texas Children’s Hospital, Baylor College of Medicine, Houston, Texas USA; 11 Department of Clinical Radiology, Great Ormond Street Hospital for Children NHS Foundation Trust, Great Ormond Street, WC1H 3JH London, UK; 12 UCL Great Ormond Street Institute of Child Health, London, UK; 13 NIHR Great Ormond Street Hospital Biomedical Research Centre, London, UK; 14 Department of Clinical Radiology, St. George’s Hospital, London, UK

**Keywords:** Artificial intelligence, Children, Education, Machine learning, Radiology

## Abstract

A new task force dedicated to artificial intelligence (AI) with respect to paediatric radiology was created in 2021 at the International Paediatric Radiology (IPR) meeting in Rome, Italy (a joint society meeting by the European Society of Pediatric Radiology [ESPR] and the Society for Pediatric Radiology [SPR]). The concept of a separate task force dedicated to AI was borne from an ESPR-led international survey of health care professionals’ opinions, expectations and concerns regarding AI integration within children’s imaging departments. In this survey, the majority (> 80%) of ESPR respondents supported the creation of a task force and helped define our key objectives. These include providing educational content about AI relevant for paediatric radiologists, brainstorming ideas for future projects and collaborating on AI-related studies with respect to collating data sets, de-identifying images and engaging in multi-case, multi-reader studies. This manuscript outlines the starting point of the ESPR AI task force and where we wish to go.

## Introduction

Artificial intelligence (AI), which includes machine learning and deep learning, is defined as “the theory and development of computer systems and algorithms able to perform tasks normally requiring human intelligence” [1]. This statement covers a wide range of tools including, but not limited to, visual perception, speech recognition and decision-making. A description of how AI works is “a set of advanced computational algorithms that learn patterns in data to help make predictions on unseen data sets” [2].

There has been much hype associated with AI, mainly due to the great potential it promises in many aspects of health care from improving workflow, automating mundane tasks, early recognition and prediction of common, as well as rare, potentially treatable diseases and better usage of scarce resources [3]. Whilst the medical literature has seen an exponential rise in publications asserting the superhuman performance of AI algorithms, this has yet to translate into wider clinical practice. Nevertheless, most leaders in the field believe it will be only a matter of time before they become commonplace [4], with most general radiologists welcoming this innovation [5–8].

As a response to this growing interest in AI, an international survey canvassing health care professionals in paediatric radiology was conducted on behalf of the European Society of Paediatric Radiology (ESPR) [9] to address whether similar attitudes were shared in our subspecialty. Most respondents had a positive outlook regarding integration of AI tools for paediatric radiology. Most did not believe AI would threaten their livelihood and could understand and suggest different ways in which it might be helpful for patients and general workflows. Many believed it was very important (score of 10/10) for radiology departments and hospital staff to start preparing for AI and machine learning tools, although local barriers (e.g., time, funding and availability of guidance/mentorship) were holding them back from educational opportunities.

Of the respondents who identified as ESPR members, 83% (67/81) supported a dedicated AI task force, which led to a new committee established at the International Paediatric Radiology (IPR) conference in 2021 in Rome, Italy, to accommodate this growing interest and to provide a forum where like-minded interested individuals could discuss new ideas and share learning and research opportunities. This manuscript illustrates the starting point of the ESPR AI task force and addresses our key objectives and plans.

### Task force functions

The role of AI in paediatric imaging is still emerging [10]. Whilst there are several novel uses for AI within radiology applied to adults and an increasing number of commercially available products entering a progressively crowded marketplace, few are tailored to the paediatric population [3]. New rules and regulations concerning safety and legal standards across different nations are important for these medical devices to follow [11, 12], bringing with them ethical considerations not previously addressed in other medical domains [13]. As such, it is likely that the function and goals of this task force may evolve along with the developments in the field. At the time of writing, however, the consensus view of this committee regarding its primary role and function is based upon collaborative and educational efforts, as highlighted from our members [9] and outlined below.

### Objective 1: education and training

A training programme dedicated to understanding the basic principles, applications and terminologies surrounding development, testing, implementation and safety of AI tools is needed. This will empower radiologists to understand the available literature on this topic, engage with multicentric studies to develop AI solutions and have meaningful discussions with vendors of AI tools when considering whether to integrate these into their own clinical practice [14].

We acknowledge that most radiologists do not have a background in computer science, informatics or coding, and will not see it as their role to acquire this knowledge; nonetheless, it is every doctor’s responsibility to be engaged with new developments within our subspecialty and to put patients’ outcomes and safety first. Our aim will therefore be to ensure all educational material has a clinical relevance that can be demonstrated rather than focusing on seemingly technical, mathematical or engineering-heavy topics.

In order for AI and machine learning advancements to be effectively utilised in clinical care, it is necessary for radiologists to be prepared to handle these changes. A foundational understanding of this incorporated into radiology teaching curriculums with possible involvement of university partners may be the longer-term key goal. Courses teaching the basics of AI exist but are usually offered as a single one-off event [15], institution specific (only for local staff) or aimed at attendees with some coding experience. In the international survey of opinions regarding AI in children’s imaging [9], it was interesting that more than half (68%) of respondents were more likely to learn about it if it was specifically tailored to paediatric radiology. This task force, therefore, believes it is important that dedicated material for paediatric radiologists should be provided and is already making plans for a training strategy in collaboration with representatives from other international imaging informatic society counterparts (the Society of Imaging Informatics in Medicine [SIIM] and the European Society of Medical Imaging Informatics [EuSoMII]) to foster network ties, promote mutual understanding and share areas of good practice.

In the meantime, we would like to direct readers interested in furthering their paediatric radiology AI knowledge to excellent articles previously published within Pediatric Radiology [16], including the recent special issue on this topic (published 2021) [10, 17–26]. Educational courses on AI and informatics for general radiology are organised by the European Society of Radiology (ESR), such as a dedicated foundation course for senior radiology trainees [27]; for those wanting some recognition of their training, a recently launched AI certificate programme from the Radiological Society of North America (RSNA) is available covering foundational knowledge in the use of AI products for clinical practice [28].

### Objective 2: research collaboration

There needs to be a general understanding that AI can play a pivotal role in medical care, and that through collaboration and sharing of multicentric data we can help validate and establish the performance of novel algorithms for paediatric use. Many of the publications describing AI algorithms fail to conduct external validation or create tools based on very biased data sets [29], which could potentially exclude certain populations (i.e. children younger than 2 years or those affected by rare diseases). There is also a lack of data and information regarding post-market surveillance and whether AI performance maintains similarly high accuracy and standards with time. To overcome these issues, we aim to share experiences of streamlined ways of working and sharing large data sets among hospitals, whether it is related to assisting in setting up federated learning databases at local sites or a secure cloud-based method of accessing anonymised information to deploy and evaluate the performance of the algorithms.

### Objective 3: collaboration for AI implementation

There is a clear need for collaboration among interested professional groups to form a strong clinical, radiologic and data/computational scientist support network. Advanced technologies and AI solutions are only as strong as the people who drive and support them. Their development depends on a user-centric design that aids health care outcomes, with proactive involvement of the end users and those who potentially benefit from their input. Partnerships and collaborations are key to delivering innovative solutions that optimise patient care. We will therefore develop and support partnerships amongst paediatric radiologists across multiple centres and subspecialties along with adult radiology colleagues (who may have some domain experience from their own practice to share), expertise from computational and engineering sciences (including software engineers and statisticians), assistance from implementation scientists, Picture Archiving and Communication System (PACS) teams and hospital managers regarding integration into clinical workflow and research partnerships.

We should also bear in mind that many stakeholders may work with or for commercial/industrial AI partners who also provide their own key insights into how to produce and incorporate their AI tools into health care. By combining these skill sets and harnessing the expertise from each domain, we can assist colleagues wishing to understand how to approach the integration and adoption of AI into their clinical department.

### Current projects

Given our above stated objectives, we have put together an ambitious yet achievable set of goals we will be working toward across the next five years that broadly cover three main domains:

- Collaboration, partnerships and support
  1. Assimilate a core group of international paediatric radiologists with an interest and expertise in using, teaching and developing AI tools.
  2. Establish a network that enables collaboration of research material (e.g., external validation data sets, imaging readers, data labelers) to support the development of AI tools and evaluation of emerging AI products for children. This might involve promoting standardization of imaging protocols to reduce the variability of examinations, to promote a high imaging quality and ultimately to improve the accuracy and performance of algorithms and radiologists.
  3. Advise and support new centres wishing to adopt AI into their clinical workflow by collating a database of ESPR members’ user implementation/experiences.
  4. Provide ESPR members with the tools and knowledge to critically analyse the AI models they use in their practice and to develop the confidence to thoughtfully question a model’s output when it does not align with their clinical intuition or experience.
- Research, ethics and data sharing
  5. Brainstorm research study ideas, identify key partners and task force members to support these projects and algorithm development.
  6. Debate the ethics of using AI for children regarding how, when, why and for whom this new technology is appropriate bearing in mind any unintended consequences for usage in paediatric disease.
  7. Collate data and publish systematic reviews on the effectiveness of AI for different paediatric diseases to keep our ESPR members up to date on the latest technology.
  8. Advise and support members on topics regarding data security, best practice methods for sharing data, de-identifying images and how to best perform external validation on AI tools for individual centres.
- Education and training
  9. Provide information for junior trainees wishing to undertake informatics training and AI experience as part of a paediatric radiology fellowship.
  10. Establish teaching sessions from internationally renowned leaders in the field of AI for paediatric radiologists at our task force meetings during the annual ESPR conference events and postgraduate courses. This will involve collaboration with other radiologic societies such as the European Society of Radiology (ESR), the European Society of Medical Imaging Informatics (EuSoMII), the Society for Pediatric Radiology (SPR), the Society of Imaging Informatics in Medicine (SIIM) and the Radiological Society of North America (RSNA). Representatives from these societies will be invited to join our task force to provide updates and guidance on news and events from other societies.

## Conclusion

The coronavirus disease 2019 (COVID-19) pandemic has brought to the forefront the need for innovative technology solutions to improve patient care. Technology does not work in isolation as it takes clinical expertise and partnerships to turn technological innovations into patient-centric solutions. We are a task force dedicated to AI in paediatric radiology and exist to serve the members of ESPR by supporting them in educational, research and collaborative outputs as well as sharing user experiences in this fast-paced and rapidly developing field.
